# Probiotics: Positive Effects on COVID-19 Patients

**DOI:** 10.21315/mjms2022.29.4.16

**Published:** 2022-08-29

**Authors:** Le Pham Tan Quoc

**Affiliations:** Institute of Biotechnology and Food Technology, Industrial University of Ho Chi Minh City, Ho Chi Minh City, Vietnam

Dear Editor,

Nowadays, there has been the appearance of some new coronavirus variants, including Delta and Omicron, and the Coronavirus disease 2019 (COVID-19) pandemic has spread dramatically all over the world. In many developing countries, the shortage of vaccines has become serious, with a large number of patients with severe disease. According to Maxmen (1), less than 1% of people in low-income countries are fully vaccinated, while in lower-middle-income countries, this proportion is only 10%. However, fortunately, scientists have found many solutions, besides vaccines, that could potentially prevent COVID-19 infection, although their efficiency cannot be equal to that of vaccines. But this is also the highlight of medicine.

Among them, probiotics have been proposed as potential factors for enhancing results. Probiotics are a combination of live beneficial bacteria and/or yeasts that naturally live in the human body. They help keep your gut healthy, boost the immune system, destroy the bad bacteria and maintain the pH of the digestive system (2). In addition, probiotics also possess many other benefits for human health, such as having anti-pathogenic, anti-diabetic, anti-obesity, anti-inflammatory, anti-cancer, anti-allergic and angiogenic activities (3). The use of probiotics is also simple, safe, cheap and intrinsic. They are quite convenient for patients compared to other drugs.

According to Li et al. (4), probiotics could positively treat COVID-19 patients, decreasing secondary infections and moderating immunity. This finding is consistent with a previous study by Santacroce et al. (5), who also indicated that probiotics could strengthen and modulate the immune system against diseases. The functions of probiotics are to provide the balance in a diversified intestinal ecosystem to prevent the severe acute respiratory syndrome coronavirus 2 (SARS-CoV-2) infection. Based on these results, I wish that probiotics may be considered as a guide to the clinical therapy of COVID-19 in the future. Besides, as we have known, the mechanism of pathogenic activity of the virus on host cells occurs through entry via spike proteins (SPs) and angiotensin-converting enzyme 2 (ACE2) receptor proteins. Anwar et al. (6) proved that plantaricin synthesised from *Lactobacillus plantarum* can block entry of the virus by binding with ribonucleic acid (RNA) dependent RNA polymerase, residual binding protein and ACE2. The blocking of major structural SPs has an important function in the life cycle of the virus and this can possibly be one of the best targets for other molecules.

In fact, probiotics possess some advantages for patients through several different mechanisms. However, their efficacy significantly depends on different probiotic bacterial species and strains. Thus, in my opinion, probiotics are not strange for scientists, but they still contain many mysteries for medicine to discover. We need more research in this area to improve knowledge and apply it widely in the pharmaceutical and medical fields in the current period and maybe deal with bad problems in the future.
